# National Birth Defects Prevention Month and Folic Acid Awareness Week — January 2014

**Published:** 2014-01-03

This year, National Birth Defects Prevention Month focuses on how common, costly, and critical birth defects are in the United States. Birth defects are relatively common, affecting one in every 33 infants born in the United States each year, or approximately 120,000 infants (1). Birth defects also are costly. Each year, total hospital costs for U.S. children and adults with birth defects exceed $2.6 billion, not including costs for outpatient care or many provider charges (2). As the leading cause of infant mortality, birth defects also are critical, accounting for one in every five infant deaths (3).

January 6–12, 2014, is National Folic Acid Awareness Week. If a woman consumes the recommended amount of folic acid before and during early pregnancy, it can help prevent major birth defects of the brain and spine (neural tube defects) (4). Health-care providers should encourage every woman of childbearing age to consume folic acid from fortified foods or supplements, or a combination of the two, in addition to a varied diet rich in folate. Additional information about folic acid is available at http://www.cdc.gov/folicacid.

Health-care professionals can help prevent many other birth defects by encouraging women of childbearing age to manage health conditions and adopt healthy behaviors before becoming pregnant, including not drinking alcohol (5) or using tobacco (6), controlling their blood glucose if they have diabetes (7), maintaining a healthy weight before becoming pregnant (8), and limiting prescription and over-the-counter medications to those that are essential (9). Additional information is available at http://www.cdc.gov/birthdefects.
